# Enhanced Depth Navigation Through Augmented Reality Depth Mapping in Patients with Low Vision

**DOI:** 10.1038/s41598-019-47397-w

**Published:** 2019-08-02

**Authors:** Anastasios Nikolas Angelopoulos, Hossein Ameri, Debbie Mitra, Mark Humayun

**Affiliations:** 1 University of Southern California, USC Ginsburg Institute for Biomedical Therapeutics, Los Angeles, 90033 USA; 2 Stanford University, Department of Electrical Engineering, Stanford, 94301 USA; 3 University of Southern California Keck School of Medicine, USC Roski Eye Institute, Los Angeles, 90033 USA

**Keywords:** Translational research, Software, Biomedical engineering

## Abstract

Patients diagnosed with Retinitis Pigmentosa (RP) show, in the advanced stage of the disease, severely restricted peripheral vision causing poor mobility and decline in quality of life. This vision loss causes difficulty identifying obstacles and their relative distances. Thus, RP patients use mobility aids such as canes to navigate, especially in dark environments. A number of high-tech visual aids using virtual reality (VR) and sensory substitution have been developed to support or supplant traditional visual aids. These have not achieved widespread use because they are difficult to use or block off residual vision. This paper presents a unique depth to high-contrast pseudocolor mapping overlay developed and tested on a Microsoft Hololens 1 as a low vision aid for RP patients. A single-masked and randomized trial of the AR pseudocolor low vision aid to evaluate real world mobility and near obstacle avoidance was conducted consisting of 10 RP subjects. An FDA-validated functional obstacle course and a custom-made grasping setup were used. The use of the AR visual aid reduced collisions by 50% in mobility testing (p = 0.02), and by 70% in grasp testing (p = 0.03). This paper introduces a new technique, the pseudocolor wireframe, and reports the first significant statistics showing improvements for the population of RP patients with mobility and grasp.

## Introduction

**Figure 1:**
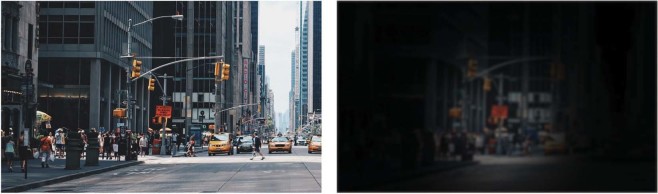
Simulation of view of patient with advanced stage Retinitis Pigmentosa. Notice the smaller field of view, lower visual acuity, and lower brightness. The individual experiences of RP patients vary widely; some, for example, have peripheral islands of vision, and many have degraded color vision.

One to three million people worldwide have Retinitis Pigmentosa (RP)^1–3^. RP is an inherited retinal disease, in which cone and rod photoreceptors are progressively lost, often leading to blindness. Typically, patients with RP experience dark adaptation issues and night blindness in adolescence, lose peripheral vision in young adulthood, and lose central vision later in life^4,5^. RP patients, due to the low field of view during advanced stages of the disease (illustrated in Fig. 1), need assistive devices (e.g. canes) to complete basic tasks such as mobility. Also, patients with pigmentary retinopathy, which “mimics” RP, and syndromes such as Usher’s Syndrome, in which RP is a symptom, suffer from the same challenges^6,7^.

Original used with permission^8^.

RP patients, especially in advanced stage, struggle with mobility and collide with obstacles at five times the normal frequency in low light^9–11^. They also have poor dark adaptation and object grasping capabilities^12,13^. This hinders the daily lives of visually impaired people as they struggle to perform basic tasks, like seeing in changing light conditions, navigating in unfamiliar places, walking outdoors, and engaging in leisure activities^14^. Unsurprisingly, visual field (VF) loss has a strong inverse correlation with vision-related quality of life using standard metrics like the National Eye Institute Visual Function Questionnaire-25^15^. Many people with RP experience anxiety and “devastation” at the thought of losing their independence and freedom of movement^16^.

Wearable electronic low-vision aids purport to improve mobility and basic task performance by helping RP patients determine the location and distance of objects from their body. Dozens of such aids aim to supplement the low bandwidth of an impaired eye with sensory stimuli^17–29^. Although useful to some extent, aids that use virtual reality (VR)^18–20^, auditory feedback^18,21–23^, and haptic cues^24–26^ often require significant training and slow down the mobility of patients, despite improving hazard avoidance in some cases^27^. However, over time, mobility speed may also improve: Hicks *et al*., for example, is one of the few examples of a VR visual aid accompanied by a sizeable user study (n = 18); this study indicates simplified depth-based navigational aids in VR are easy to use for patients and improve collisions and time to completion over the course of 10 tests^28^. Each of the above devices has its own set of challenges: auditory and haptic cues require retraining the brain to understand complex mappings between audio/haptics and 3D space^30^, and VR occludes patients’ natural vision in favor of rendering algorithms which often magnify a scene leading to a restricted field of view and also interfere with people’s natural social interactions by covering their eyes^31^. Furthermore, low battery life, the need to be tethered to a laptop, and discomfort deter potential users. Consequently, such devices have not been widely adopted by people with low vision.

Researchers have recognized these issues and proposed modifications using AR which enhance the natural senses rather than supplanting them^17^. One promising solution uses auditory Augmented Reality (AR) to sonify important 3D objects with natural language to improve navigation and object localization^32–34^. Another overlays 10 high-contrast bands of color on top of vision to improve edge detection but has yet to be evaluated for real-world mobility improvement in visually impaired patients^35^. As commercial AR headsets improve, visual aids using multiple electronic sensor inputs and object identification^18,36^ algorithms will merit further study. Younis *et al*., for example, developed a promising AR system which performs object detection, tracking, and classification to create a visual AR “warning system” for patients with low visual fields^29,37^. However, it was never tested on people, and because it relies on object categorization outside the visual field, it would require real-time eye tracking on a large field of view to be effective. Still, for people with some remaining vision who struggle with mobility and object localization, a sophisticated visual AR overlay which helps interpret the full environment (rather than only the objects classified as hazards) may support their remaining visual system sufficiently to improve functionality on basic tasks such as navigation and grasp. Furthermore, any such aid must be evaluated carefully with metrics that correspond to real-world mobility and grasp outcomes.

We have developed a novel AR pseudocolor encoding system for enhanced depth navigation: a 4-color depth encoded wireframe that can be used with commercially available AR devices. To our knowledge, this paper is the first to show a statistically significant mobility improvement when RP patients use a visual AR low vision aid in a test validated by the FDA to correspond with a real-world mobility improvement, and also the first to do the same in a grasp experiment.

## Results

Ten RP subjects with VA < 20/80 or VF < 30° performed a highly controlled, reconfigurable obstacle course with AR on or off in a random order (i.e. for the first trial, some had the depth encoding enabled and some had it disabled to avoid learning effects). Using an anonymized video of each test, a masked grader recorded the number of times a person collided with obstacles and also the time it took each subject to complete the course (see Methods section for details of masking). We found that with AR on, RP subjects make significantly fewer errors in mobility and grasp tasks (50% with p = 0.02, 70% with p = 0.03). No significant result was reached regarding time to completion.

Prior to testing with RP subjects we completed preliminary experiments on twelve sighted subjects wearing constricted field of view glasses, finding a significant reduction in mobility and static grasp collisions (66%, p = 0.005 and 68–85%, p = 0.03). The simulation glasses correctly simulated small VF, but did not, for example, degrade color vision or central vision as can often be the case in RP. The subjects were recruited prior to and independently of the RP experiments as a proof of concept. These experiments are described in Supplementary Section 1 and suggest mobility can improve generally for patients with small VF.

### Mobility Results

#### Decrease in Collisions: Mobility

**Figure 2:**
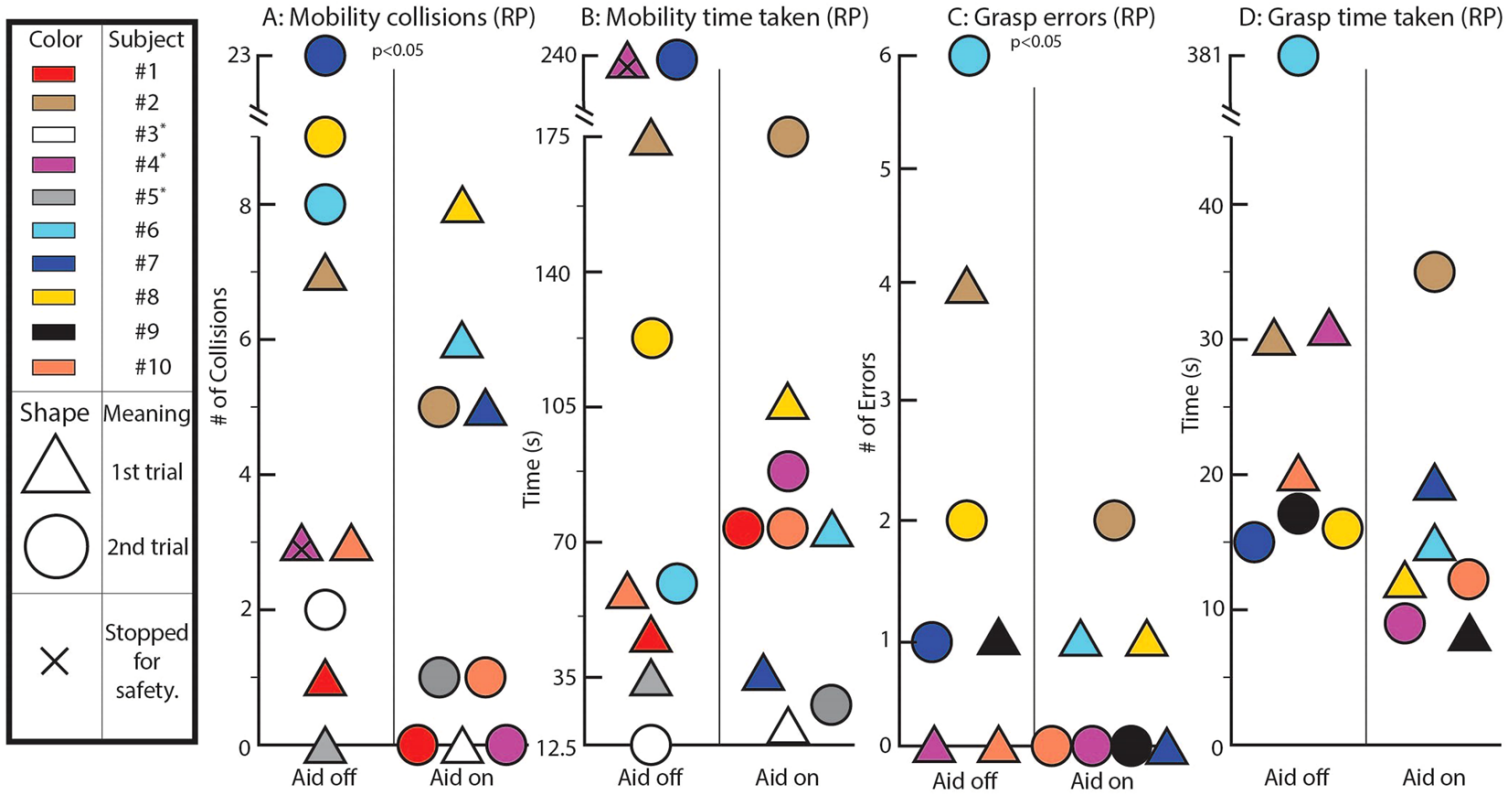
Results from RP subjects. In these plots, each color corresponds to a different subject. “1st trial” and “2nd trial” refer to whether the subject was tested with AR first or second temporally; for example, subjects with a triangular shape in the “Aid on” column were tested with the pseudocolor encoding on first. The vertical axis is either the number of collisions during course performance or the time taken to complete the course. The horizontal axis, which has two discrete values, denotes whether AR was off or AR was on for that trial. (**A**) Number of collisions during obstacle course testing of eligible subjects with RP. (**B**) Time taken to complete obstacle course testing by eligible RP subject. (**C**) Number of errors (misidentification, collision, etc.) during RP grasp experiment. (**D**) Time taken to complete grasp experiment by eligible RP subject. An expanded version of this figure with bar charts is included as Supp. Fig. 5 for interpretability.

RP subjects had on average 50% fewer collisions with AR on as opposed to AR off (Fig. 2A, p = 0.02). Eight of the nine subjects performed better with AR on.

#### Time to Completion: Mobility

With AR on, RP subjects had a 30% shorter time to completion, but this was not statistically significant. One subject, #5, was unable to complete the obstacle course without the device. Another subject, #9, was unable to identify the endpoint of the maze for four minutes. The result was not significant, with or without normalization based on the subjects’ preferred walking speed (Fig. 2B).

### Grasp Results

#### Decrease in Collisions: Grasp

Of the four RP subjects who made grasp errors, each made significantly fewer errors with AR than without. This improvement constituted a 70% increase in grasp performance (p = 0.03) (Fig. 2C).

#### Time to Completion: Grasp

Four of the seven RP subjects completed the grasp experiment in less time with AR than without. The mean improvement was 78% but it was not statistically significant (p = 0.09, Fig. 2D).

## Discussion

Subjects collided with fewer obstacles with the help of AR pseudocolor in a clinically validated obstacle course with a variety of object sizes and contrasts. All data from every enrolled RP subject are reported in Fig. 2, and only one subject outperformed AR when unaided. As Chung et. al. show, this performance should correspond to increased safety, comfort, and ease of real-world mobility in many low-light environments from restaurants to movie theaters to the great outdoors^38^. Subject 4, for example, reported difficulty walking home from work in the evenings, and routinely tripping over curbs and into bushes. Each subject has a wide range of mobility difficulties, exacerbated after twilight. Subject 9 reported avoiding going out almost every evening due to fear of falling, harming their emotional wellbeing. Such lived experiences are consistently reported by RP subjects^14^. Because the proposed AR aid helps improve subjects’ mobility particularly in low-light scenarios, it may profoundly improve quality of life. Similarly, the reported increase in grasp performance may improve confidence in using utensils, household appliances, and other basic tasks.

This paper uses a rigorous experimental procedure to negate limitations, ensure reproducibility, and mitigate other areas of bias. Starting with the selection of three clinically validated obstacle course configurations, the randomized trial methodology averages out any maze difficulty and learning effects; the randomized, masked grading system removes personal bias; and the structured, audio-guided training procedure ensured consistency in training. All experiments are outlined in detail and audio training procedures are included in Supplementary Data files 1 and 2. Further careful validation will be a necessary step in the clinical implementation of this technology, and these experiments are an important first step establishing clinical utility of AR pseudocolor.

Augmented Reality visual enhancements for low vision mobility have been suggested as an alternative or supplement to traditional low vision aids such as the cane or the guide dog for almost two decades^39^. Hicks *et al*., in a VR visual aid study, showed significantly improved navigational skill with a simplified depth encoding in grayscale; as future work, they suggest that since people with visual impairments are very skilled at identifying objects with residual vision, an AR approach may provide even more benefit^28^. Furthermore, the methodology of Hicks *et al*. could be improved by a clinically validated mobility test which controls for illumination, object contrast, learning effects, etc. Still, studies like Hicks *et al*. are useful precursors for AR visual aids. AR aids pre-Hololens relied on proprietary hardware implementing computer vision algorithms in real time^40,41^. More recently, since the development of commercial AR headsets, these methods seem ever-more feasible. For example, Coco-Martin *et al.* use binocular disparity to calculate depth and encode it as color along depth edges and showed in some preliminary experiments that the device may preserve the preferred walking speed (PWS) of RP subjects^42^. However, the system they developed is proprietary and does not take advantage of state-of-the-art 3D reconstruction methods implemented on commercial AR devices like the Microsoft Hololens. Consequently, depth can only be rendered onto edges, making it difficult for RP patients with already low fields of view to identify the surfaces of obstacles. At the same time as we were performing our experiments, Kinateder et. al. performed an “exploratory study” on four visually impaired people with three different etiologies but used a mobility metric that is neither realistic nor clinically validated: measuring the distance at which a subject first recognizes an obstacle^35^. Thus, prior work indicates that AR may be useful, but fails to optimize a low-vision aid for any particular etiology, show significant results indicating that it will help a population of blind people with mobility, or use a clinically meaningful methodology. Furthermore, there has been no discussion of grasp in AR.

This study builds on the prior work by providing statistically significant mobility and grasp improvements in a population of 10 RP subjects using a methodology based on an FDA-validated clinical study. This study also introduces new technical methods for AR low vision aids: (1) The pseudocolor wireframe is designed to help Retinitis Pigmentosa patients even with significant color deficiencies, and shows that a very coarse color-to-depth map improves mobility even in individuals with very poor color vision; (2) Rather than continuously rendering a surface over the real world, we construct a triangular point mesh using a geometric shader, which preserves the abilities of individuals to perform tasks such as reading text with their normal remaining vision; (3) our wireframe does not go farther than 6 feet (as opposed to infinity), preventing sensory overload and increasing user comfort and wireframe interpretability; and (4) this paper optimizes AR for the specific visual characteristics of RP, and has strong significant results indicating high levels of visual confidence, interpretability, and intuitiveness. This methodology, of designing rendering techniques for specific etiologies of blindness, is promising for future aids, given the results. The improvements in collision rate and depth discrimination arise both from the explicit depth-to-color mapping and from the increase in brightness/contrast provided by AR.

Other intuitive forms of depth encoding should also be studied, such as time-domain oscillation of the brightness, saturation of high-risk objects, audiovisual cues, and tracking the velocity and depth of objects to determine their risk to the subject. Eye tracking should also be explored, as subjects may not be able to see visual warning signs due to restricted field if they are looking at the wrong part of the screen. A robust aid could incorporate eye tracking to warn users with directional sound if they cannot see an obstacle and highlight it when they look. Such an eye-tracked aid, which would be possible with the Hololens 2, could thereby take a user’s visual field into account both for aid effectiveness and also rendering efficiency.

Future studies to address mobility improvement with higher training and usage time are warranted given the parameters and limitations of this study. The reason we did not achieve significance in time to completion is likely because subjects had not acclimated to the use of the device and we only tested each subject in the obstacle course 2–3 times, in a randomized order, and after extremely limited training. This effect is consistent with previous studies on VR visual aids. Van Rheede *et al*. quantitatively show that with low training time, their VR visual aid increases hesitation and lowers walking speed; however, they claim that this effect disappears over time^43^. Correspondingly, Hicks *et al*. show in a user study that after 10 maze trials, subject time to completion is cut in half, while after only one trial, the difference in time to completion is not significant^28^. Our results are consistent with these investigations. Based on these we would expect improvement in time to completion with prolonged use. Future work should quantify how much training is necessary to achieve a benefit in time to completion.

Another limitation of this study is the lack of intra-grader reliability assessment. However, the reviewer was a doctor who was trained in an orientation for how to grade videos. We based our study on Chung *et al*., which reported an inter-grader reliability of 98% when testing every 3 months; we had only one grader who graded all videos in less than a week, so we expect to have a similarly high reliability^38^.

On the technological level, general limitations of Augmented Reality as a low vision aid include poor real-time depth mapping, small field of view, limited battery life, weight, PC tethering, and high cost. Real-time Simultaneous Location And Mapping (SLAM) should be incorporated out of the box in AR systems for dynamic environments, but the Hololens 1 only updated every second. Small field of view forces subjects to crane their heads down to see obstacles. The battery life of the Hololens 1 and 2 are both roughly 2–3 hours, which is not enough time to make it through a workday. Because of the intensive compute requirements of AR, the Hololens and other untethered devices suitable for mobile use are quite heavy. Finally, these devices cost thousands of dollars ($3500 for the Hololens 2), making them inaccessible to patients. If all the above limitations are solved by AR companies, then AR will be a much more effective platform for mobility and grasp aids.

In conclusion, this paper advances the state of this field because, to our knowledge, it is the first study to do three things: (1) Show a statistically significant mobility improvement for patients with RP using a visual AR low vision aid in a test validated by the FDA to correspond with a real-world mobility improvement. (2) Demonstrate a new low vision aid technique, the pseudocolor wireframe. (3) Validate the ability of AR to improve grasp in patients with RP. In total, the contribution of this paper is a new low vision AR methodology (the wireframe), and a tightly-controlled and highly reproducible experiment which shows it can improve the mobility and grasp of subjects with a methodology relevant to clinical and real-world application.

## Methods

### Device and method of encoding depth

**Figure 3:**
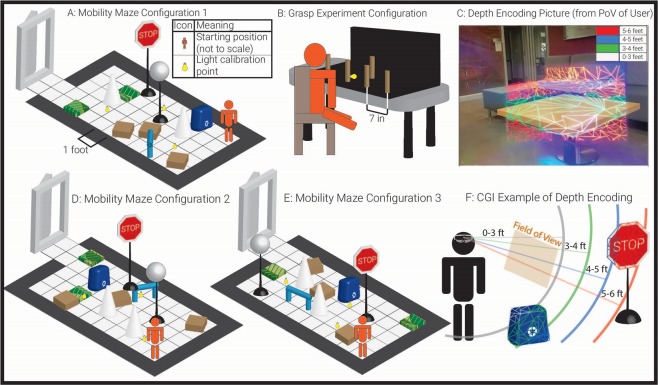
Experimental setup and images of pseudocolor depth encoding. (**A**) Experimental course configuration one. (**B**) Grasp experiment setup, with five wooden cylinders and a black backdrop. Subjects are asked to grab the wooden cylinder in the rear without touching the ones in the front. (**C**) A monocular picture, from the user’s point of view, of the pseudocolor encoding. Shows limited field of view. Image artifacts were produced by cell phone camera. (**D**) Experimental configuration two. (**E**) Experimental configuration three. (**F**) CGI rendering of pseudocolor encoding. In the mobility experiments, the coarse color map (0–3 ft = red, 3–4 ft = green, 4–5 ft = blue, 5–6 ft = red) was used. However, in the grasp experiments, the color map was finer (0–6 in = white, 6–12 in = green, 12–18 in = blue, 18–24 in = red, not shown).

The device used was a Microsoft Hololens 1^44^ and the distance encoding was a form of pseudocolor, or false-color, which mapped depth to four discrete color changes (Fig. 3C,F). The Microsoft Hololens 1 was chosen based on well-documented spatial mapping software in the Microsoft Mixed Reality Toolkit (MMRT), and the fact that it is a stand-alone, freely mobile unit which does not need to be connected to a PC^45^. Testing was performed in Summer 2018, before the Hololens 2 was announced. Using Unity and the MMRT, a triangular polygon mesh was overlaid on top of natural vision and color-coded based on distance to the user (Fig. 3C,F). In the mobility mode, objects were colored white when an object was less than 3 feet away from the headset, green when objects were 3–4 feet away, blue when objects were 4–5 feet away, and red when objects were 5–6 feet away. Objects farther than 6 feet away were not colored. In the grasp mode, objects were colored white at 0–6 inches away, green at 6–12 in, blue at 12–18 in, and red at 18–24 in. We coded these distances into a geometric shader, then verified their accuracy with a tape measure. Mathematically, points in the triangular mesh and their connectivity are given by the Hololens’ internal SLAM algorithm (through the MMRT); we take the edges of the graph and color them by calculating the distance along each line to the user with linear interpolation. The concept can be implemented in many ways, agnostic of rendering details such as the type of mesh or rendering software used. This is enabled easily by Unity or any other rendering engine. The precision of the Hololens was within one centimeter, with a 6.64% error rate. Microsoft has not released information about the smallest object the Hololens can register, as this is also function of the object contrast, lighting, and distance to the object. Roughly, from head height, objects around 2–3 inches in length can be mapped. When rendered on the transparent screens of the Hololens headset, this wireframe allowed one to see the shape, color, and features of the original object as well as its color-encoded distance, as in Fig. 3C. The depth encoding was calibrated in brightness before each trial to ensure both the obstacles and the pseudocolor were simultaneously visible. A video of the encoding is included as Supplementary Data File 3.

We chose this method of depth encoding for the following reasons: (1) Though RP often severely degrades color vision, we ordered the selection of colors based on the spectral sensitivity of the human eye^46^. The most common axis of deficiency is tritanopia, so we did not include a blue-yellow edge in our mapping^47^. This is physically consistent because though RP is a rod-cone dystrophy, it affects the rods more^4^. During testing, even subjects with anarchic D-15 color test scores were able to easily distinguish between the colors displayed by the headset. (2) The decreased visual field of RP subjects necessitates a depth encoding dense enough to be seen almost at all times; if only edges were enhanced, objects with few edges would be difficult to see. Clinical results have shown that advanced RP patient mobility performance is highly dependent on contrast sensitivity. Thus, edges are very important to RP patients, so an effective aid must both enhance edges when they are in the field of view and also supplement the lack of edges when there are none in the field^48^. We use color to perform this substitution. (3) It has long been known that object color is important for edge identification and motion tracking^49,50^. A system which completely overlaps objects’ natural color would interfere significantly with these cues. Our approach splits the difference, co-opting some of the perceptual edge-enhancing properties of color for the purposes of depth detection while attempting to retain the object’s natural color which can be seen through the wireframe.

The Microsoft Hololens 1 was chosen for ease of development and mobility use, as at the time of testing, the Magic Leap One headset had not been released. Other headsets, like the Meta 2 and Epson Moverio, are tethered to a computer or smartphone, and we wanted to avoid this for mobility testing. Moreover, the Hololens has a fairly stable inbuilt SLAM algorithm compliant with Unity^51^. The Hololens had one drawback, which is a very small field of view (34°) compared to other headsets like the Meta 2 (80–90°). Consequently, subjects use head tracking while wearing the device to identify obstacles, and we had to optically align subjects by asking them if they could identify all four corners of the Hololens’ virtual screen and adjusting the headset manually. With larger field of view, eye-tracked aids could be even more effective.

### Obstacle course design

Visually impaired subjects completed two tests: an obstacle course completion test and a grasp experiment test (Fig. 3A,B,D,E). The obstacle course is similar to a functional test used in the FDA-validated Voretegene Neparvovec-rzyl clinical trial^38^.

Though scientists have designed several obstacle courses to assay low and ultra-low vision mobility^52–56^, the only obstacle course used in an FDA-validated clinical trial for RP as a functional test is described by Chung et. al. This obstacle course and accompanying methodology was the foundation for this paper’s mobility methods. Special overhead lighting controlled for luminance, and objects ranged in size, height, location, and contrast. All configurations of the course are the same length (19.6 m) when navigating the best path by straight lines. The course was modified slightly in our study, removing the requirement that subjects navigate by reading arrows and the black hole obstacles, because this was not relevant to the testing of the obstacle avoidance device. Figures 3A,D,E describe the three configurations of the obstacle course.

### Grasp experiment design

In the grasp experiments, subjects were asked to grasp a wooden peg, located 18 inches behind four other wooden pegs, without touching any of the front pegs (Fig. 3B). The four front pegs were 7 inches apart. The background was black.

### Randomization and grading

**Figure 4:**
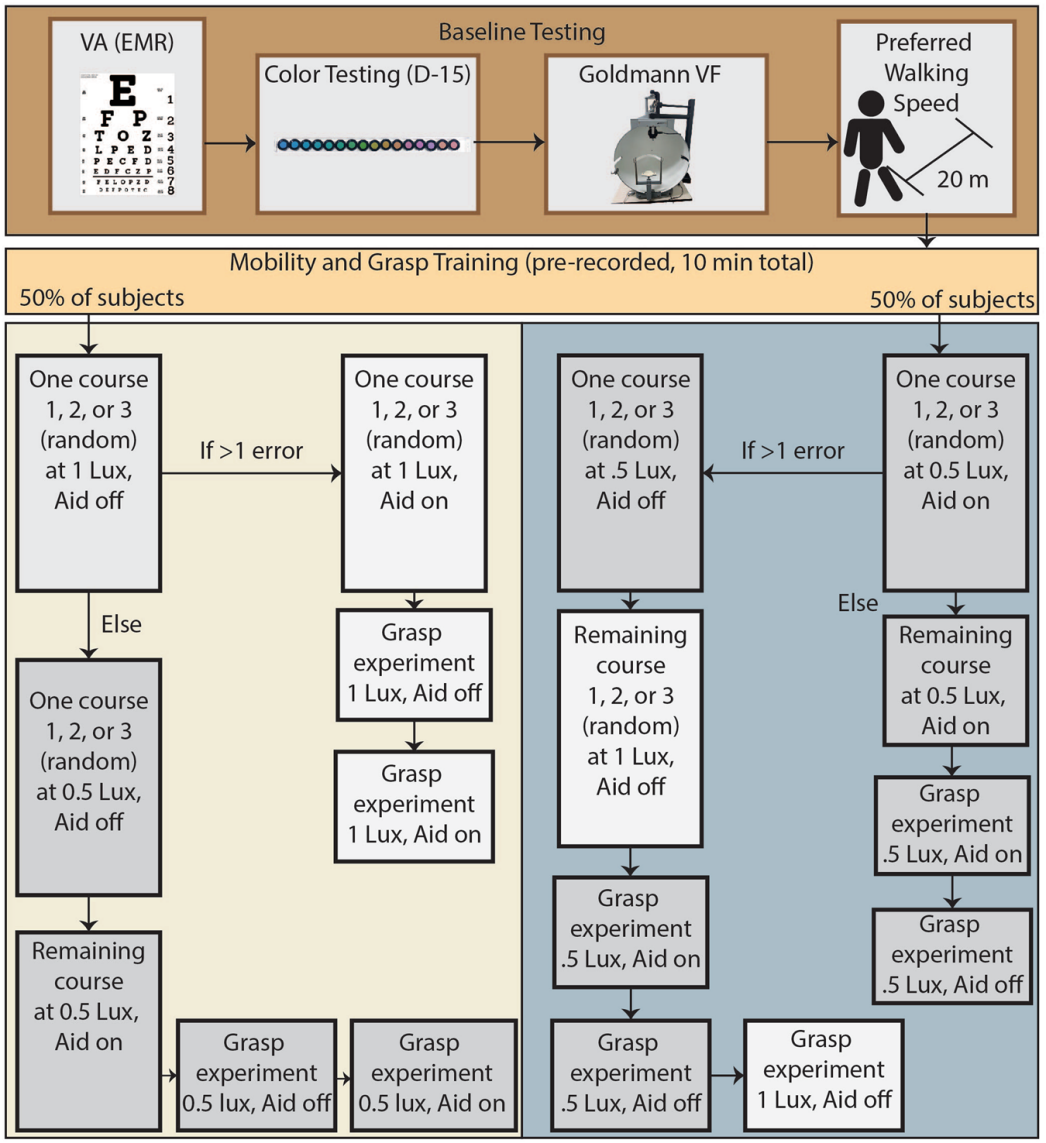
Flowchart of experimental procedure. Subjects begin by completing baseline testing, then complete a standard training sequence using audiotapes, and then proceed to one of two experimental protocols, randomizing the order of AR usage. Each subject completes course 1, course 2, and course 3 exactly once in a random order.

Data collection followed the flowchart in Fig. 4. All experiments were completed with the University of Southern California Institutional Review Board approval, in compliance with regulations, and with informed consent from all subjects. Both mobility and grasp experiments were videotaped and later graded by a single masked grader who counted errors (anytime an obstacle was touched) and did not know any information about experimental variables such as whether the device was in use or not. Mobility experiments were videotaped from two angles. Videos were given to the grader with random number generated titles and were graded in random order. The order of obstacle course administration was randomized to avoid learning effect and control for the relative difficulty of each course. The order of lighting levels was randomized. The order of device usage was randomized. Subject identity and AR device were obfuscated through Gaussian facial blur in Adobe Premiere to avoid bias. Time was started when a researcher said “Go”, and time was stopped when the subject touched the door at the end of the maze. Patients were not dark adapted before beginning obstacle course testing or grasp testing and returned to standard lighting for 10 minutes between each test. The Hololens was worn to control for its tint in all tests, and turned off or on to test the encoding.

### Clinical endpoints and eligibility

The primary clinical endpoints were: (1) Reduction in obstacle collision rate during mobility and grasp. (2) Reduction in time taken to successfully complete obstacle courses and grasp experiments. Time taken to complete these tasks is a conservative clinical indicator due to limited training. The inclusion criteria were: (1) Advanced stage RP (or any pigmentary retinopathy) with VA of <20/80 and/or VF of <30° using Goldmann IIIe4 in the better seeing eye. (2) Willingness to adhere to protocol. (3) Written consent. (4) Evaluability on mobility testing. The exclusion criteria were: (1) Inability/unwillingness to meet requirements of the study. (2) Incapability of performing mobility testing (the primary efficacy endpoint) for reason other than poor vision, including physical or attentional limitations. We did not include any subjects with optical opacification, such as visually significant cataracts or vitreous opacities, or retinal gliosis. Subjects were not be excluded based on their gender, race, or ethnicity.

### Baseline vision testing and training

A flowchart of the full training and testing pipeline is included in Fig. 4. Subjects first took a Farnsworth D-15 color vision test^57^ and a Goldmann kinetic visual field with IIIe4 stimulus (a bright white dot about 1/2° in diameter). Raw data is included in Supp. Fig. 4. Subjects were then asked to walk a 20 m hallway twice at their preferred walking speed (PWS). The second speed measurement was recorded. For the purposes of statistical analysis, each subject’s time to completion was divided by their PWS. This did not ultimately affect results. Subjects then completed an audiotape training sequence (Supplementary Data Files 1 and 2) which guided them through a training obstacle course (Supp. Fig. 3) and grasp experiment. The audiotape contained specific navigational instructions and instructions on how to interpret the pseudocolor encoding. If subjects had trouble following the audiotape (e.g. due to deafness), a researcher ensured the subject fully understood before moving on. Subjects were trained for <10 min in standard lighting.

### Analysis of subjects

All enrolled patients who met the inclusion criteria were tested, and their results reported. Before testing, the following baselines were administered: preferred walking speed, D-15 color vision test, and Goldmann visual field using a IIIe4 stimulus. Visual acuity was taken from medical records; the latest visual acuity was selected. One subject was excluded due to too large a visual field (35°) in the left eye. The rest of the subjects were included in the study. All patients, even those with extreme D-15 color deficits, were able to recognize the colors on the AR screen. Subjects 3 and 5 had to leave before grasp experiments were complete due to time constraints. Subject 4 was stopped for safety during mobility experimentation without aid and thus was assigned the same completion time as subject 7, and the raw error count was used for analysis (so, we underestimate the improvement this subject experienced with AR aid). Subject 9 did not follow instructions for the mobility experiment. In Fig. 2, these subjects are marked with asterisks. We have, in Table 1, summarized subject information, including phakia (presence of natural crystalline lens) and presence of cystoid macular edema (CME). We have included this information for completeness, but the focus is primarily on functional vision (VF, VA, color), as our references indicate that these functional vision measures are primarily responsible for mobility challenges regardless of etiology, both in heterogeneous populations and also in RP^58,59^. Subject 6 has pigmentary retinopathy, a condition known to “mimic retinitis pigmentosa,” as it has the same symptoms: “retinal dystrophic and pigmentary changes and the frequent association of night blindness, reduction of visual acuity, constriction of visual fields, and abnormal electroretinographic (ERG) findings^6^”. We note this here but refer to all subjects as RP subjects elsewhere as the conditions are exactly the same for the purposes of this study.

**Table 1:** Subject baseline data.

#	VA (OS)	VA (OD)	VF (OS)	VF (OD)	Color Vision	Notes	Phakia	CME
1	20/100 + 1	20/60 + 1	27	27	Normal	Grasp data corrupted	Phakic: OU mild senile PSC	no
2	20/800 − 1	20/800 − 1	13	7	Poor		Phakic: clear OU	no
3	20/200	20/300 − 1	12	14	Normal	Left before grasp, PR with RP history	Phakic: no mention in chart	Severe OD
4	20/150	20/250	27	27	Minor tritan	PR, cone depression, glaucoma	Phakic: OU moderate NSC	no
5	20/50 + 1	20/50 + 2	26	27	Normal	Left before grasp	Phakic: OU mild	mild OU
6	20/40 + 1	20/50 − 2	5	5	Normal	PR and glaucoma	Pseudophakic: OU	moderate OD
7	20/40 − 2	20/70	5	5	Poor		Phakic: OU	no
8	20/200 − 1	20/800	5	5	Poor		Phakic: OD NSC, Pseudo, OS	no
9	20/800	20/800	20	25	Poor	Ushers’	Pseudo, OS, Phakic: NSC OD	no
10	20/50 − 1	20/50 − 2	20	25	Minor tritan		Phakic: no mention in chart	moderate OS
S	20/20	20/20	35	40	Normal	Simulation glasses	N/A	N/A

OU, OD, and OS are medical abbreviations for both eyes, right eye, and left eye, respectively. Visual acuity was taken from last eye exam. Subjects with phakia (documented with OCT) and cystoid macular edema (CME) are noted. In two cases, no cataract was noted in chart. PSC means senile posterior subcapsular cataract, NSC means nuclear sclerotic cataract. Subjects did not have optical opacification or retinal gliosis. Field and color vision were measured on the day of testing. Full Goldmann VF and D-15 color test are included in Supp. Fig. 3.

### Statistics

A two-sided Wilcoxon signed rank test was used for all statistical findings^60^.

## Supplementary information

Supplement

## Data Availability

All data associated with this study are available in the main text or the Supplementary Materials.
