# Supplementary material for: Enhanced Depth Navigation Through Augmented Reality Depth Mapping in Patients with Low Vision
Source: Sci Rep. 2019 Aug 2;9:11230. doi: 10.1038/s41598-019-47397-w (PMC6677879; doi:10.1038/s41598-019-47397-w)
Supplement: Supplementary file 1 — Supplement [file 41598_2019_47397_MOESM1_ESM.pdf]

Supplementary Materials: Enhanced Depth Navigation Through Augmented Reality Depth Mapping in Patients with Low Vision

Anastasios Nikolas Angelopoulos[1,2,*], **Hossein Ameri**[1,3], **Debbie Mitra**[1,3], **and Mark Humayun**[1,3,*]

[1]University of Southern California, USC Ginsburg Institute for Biomedical Therapeutics, Los Angeles, 90033, USA
[2]Stanford University, Department of Electrical Engineering, Stanford, 94301, USA
[3]University of Southern California Keck School of Medicine, USC Roski Eye Institute, Los Angeles, 90033, USA
[*]nikolasa@stanford.edu, humayun@med.usc.edu

ABSTRACT

Patients diagnosed with Retinitis Pigmentosa (RP) show, in the advanced stage of the disease, severely restricted peripheral vision causing poor mobility and decline in quality of life. This vision loss causes difficulty identifying obstacles and their relative distances. Thus, RP patients use mobility aids such as canes to navigate, especially in dark environments. A number of high-tech visual aids using virtual reality (VR) and sensory substitution have been developed to support or supplant traditional visual aids. These have not achieved widespread use because they are difficult to use or block off residual vision. This paper presents a unique depth to high-contrast pseudocolor mapping overlay developed and tested on a Microsoft Hololens 1 as a low vision aid for RP patients. A single-masked and randomized trial of the AR pseudocolor low vision aid to evaluate real world mobility and near obstacle avoidance was conducted consisting of 10 RP subjects. An FDA-validated functional obstacle course and a custom-made grasping set up were used. The use of the AR visual aid reduced collisions by 50% in mobility testing (p=0.02), and by 70% in grasp testing (p=0.03). This paper introduces a new technique, the pseudocolor wireframe, and reports the first significant statistics showing improvements for the population of RP patients with mobility and grasp.

Supplementary Materials

Supplementary Section 1: Preliminary Experimental Results and Methodology on Sighted Subjects

Results:

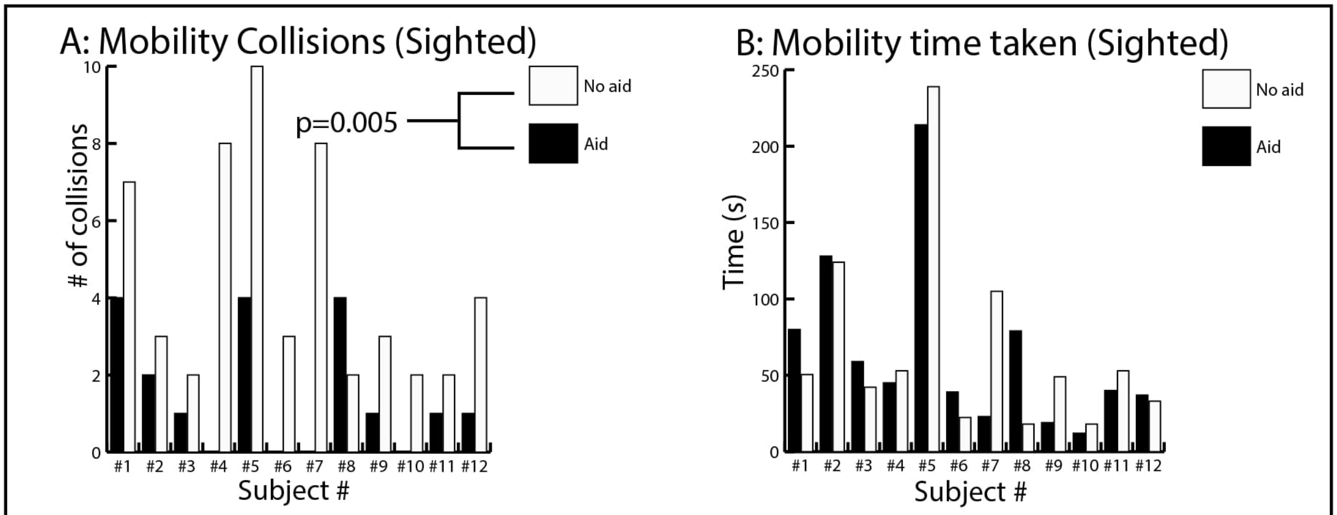

C: Table of Grasp Statistics (Sighted)

Task	Δμ, errors (%)	p-value, errors	Δμ, time (%)	p-value, time
One-Handed Grasp	85	0.03	-15	1
Two-Handed Grasp	68	0.03	-41	0.34
Dynamic Grasp	20	0.58	0	0.91

Supplementary Figure 1. Results from preliminary experiments on sighted subjects wearing RP simulation glasses. (A) In the mobility experiment sighted subjects collided with fewer obstacles when using AR (66%, p=0.005). The horizontal axis indicates a particular subject, and the vertical axis indicates number of collisions. White bars indicate unaided performance and black bars indicate aided performance. Subjects 4, 6, 7, and 10 had no errors when tested with the aid on. (B) The vertical axis here means time to completion of the obstacle course. The horizontal axis is a grouping by subject and the bar colors indicate aided vs unaided performance. (C) The table shows group statistics for each of three preliminary grasp experiments completed on simulated RP subjects.

The preliminary data indicated a 68% decrease in mean number of collisions when normal sighted subjects with constricted field glasses used AR in mobility with p=0.0051 (Supp. Fig. 1A). No improvement was measured in time to completion for any experiment (Supp. Fig. 1B). The one- and two- handed grasp experiments indicate collision improvements of 85% and 68% respectively, with p=0.03. No improvement was measured in the dynamic grasp experiment (Supp. Fig. 1C).

Methods:

In preparation for the human subject study on visually impaired individuals, two sets of experiments were performed to determine the effectiveness of AR pseudocolor encoding on sighted volunteers wearing glasses which simulated low visual field. The volunteers wore RP simulation glasses (Good-Lite VisualEyes Vision Simulator Glass, Peripheral Field Loss Simulator), which were further occluded with black cardstock to achieve a VF between 20-40 degrees (Supp. Fig. 4). Volunteers wore the simulation glasses in between the AR headset and their eyes. Subjects enrolled in this study had no visual impairments and 20/20 vision.

First, subjects were placed in the RP Simulation glasses. Then, the Hololens was worn on top of the RP simulation glasses (the simulation glasses were in between the screen of the Hololens and the user's eyes). Subjects were allowed to wear the Hololens with our visual encoding for a maximum of 10 minutes before they were brought to the testing area. Subjects were not trained in a standard manner, but rather asked to explore a space in standard office lighting with the headset. After time was up, subjects were blindfolded and led to the testing area, where they began obstacle course testing. The order of AR usage was randomized to avoid learning effects (i.e. some subjects used the encoding first and did baseline testing second). The order of

the courses was randomized to average for course difficulty. Obstacle courses were of standard length (36 ft) with 10 standard obstacles of varying sizes and colors to assay the effect of the device on collision rate and time taken to traverse a course. Time was stopped when the subject sat in the chair at the end of the course. The courses are described in Supp. Fig. 2D and 2E. Subjects start at the red dot and go to the blue dot.

Sighted subjects were also asked to perform three grasp experiments. The first experiment is described in Supp. Fig. 2A. Two thick cylindrical dowels were placed in front of the user in the geometric configuration shown in the image. Either cylinder 1 (C1) or cylinder 2 (C2) was displaced by 1 inch with respect to the other. The subject was led blindfolded to the testing table while wearing the RP simulation glasses. Then, depending on randomization, they were asked to grasp the closest cylinder with AR on or off. Similarly to the main paper, results were compared both with respect to error count (incorrect cylinder or collision) and also time to completion. Note that collisions are less clear in the context of grasp; any unsure touches, missed grabs, or fumbles were counted as errors.

The second experiment, pictured in Supp. Fig. 2B, was to test two handed coordination and spatial understanding. A rectangular bag was placed 18 inches in front of the subject and they were asked to grasp it with both hands at the same time. Time was started when a researcher said "go," at which the subject opened their eyes, and stopped when both hands firmly grasped the bag. The setup and grading protocols were the same as the one-handed grasp experiment.

Subjects were then asked, in experiment three pictured in Supp. Fig. 2C, to perform a dynamic task, where they moved objects to perform an objective. Specifically, subjects were asked to perform the action of “pouring a pitcher” of tea into a cup. The pitcher was empty, but they were asked to grasp its handle and mimic pouring it into the cup. Time was started at a researcher’s "go," and stopped when the pitcher was returned to its starting position. The setup and grading protocols were the same as the one-handed grasp experiment.

Discussion:

The decreased collisions in mobility and grasp indicated that this device would be useful for RP patients, and motivated our main study. In retrospect, sighted subjects seem to improve much more than visually impaired subjects when using the AR device; this may be a product of increased color/contrast vision in sighted subjects, even when wearing the RP simulation glasses. Refer to the main text for the explanation of the non-significance of time to completion results reported in Supp. Fig. 1B, as these subjects also had limited training time. The fact that subjects collided with fewer objects using AR than not in the static tasks but not the dynamic "pitcher" task indicates that the real-time applicability of this system is very limited, possibly due to the slow update time (around 1s) of the internal SLAM algorithm. The limitations of this study include the lack of a masked reviewer, lack of intra-grader reliability testing, and lack of clinical validation of the tests. It is unclear, for example, whether the grasp experiments are indicative of real-world performance. Furthermore, it is unclear whether the mobility tests we used correspond to real world performance. However, this data is still useful as proof of concept towards a clinically validated methodology on RP subjects.[?].

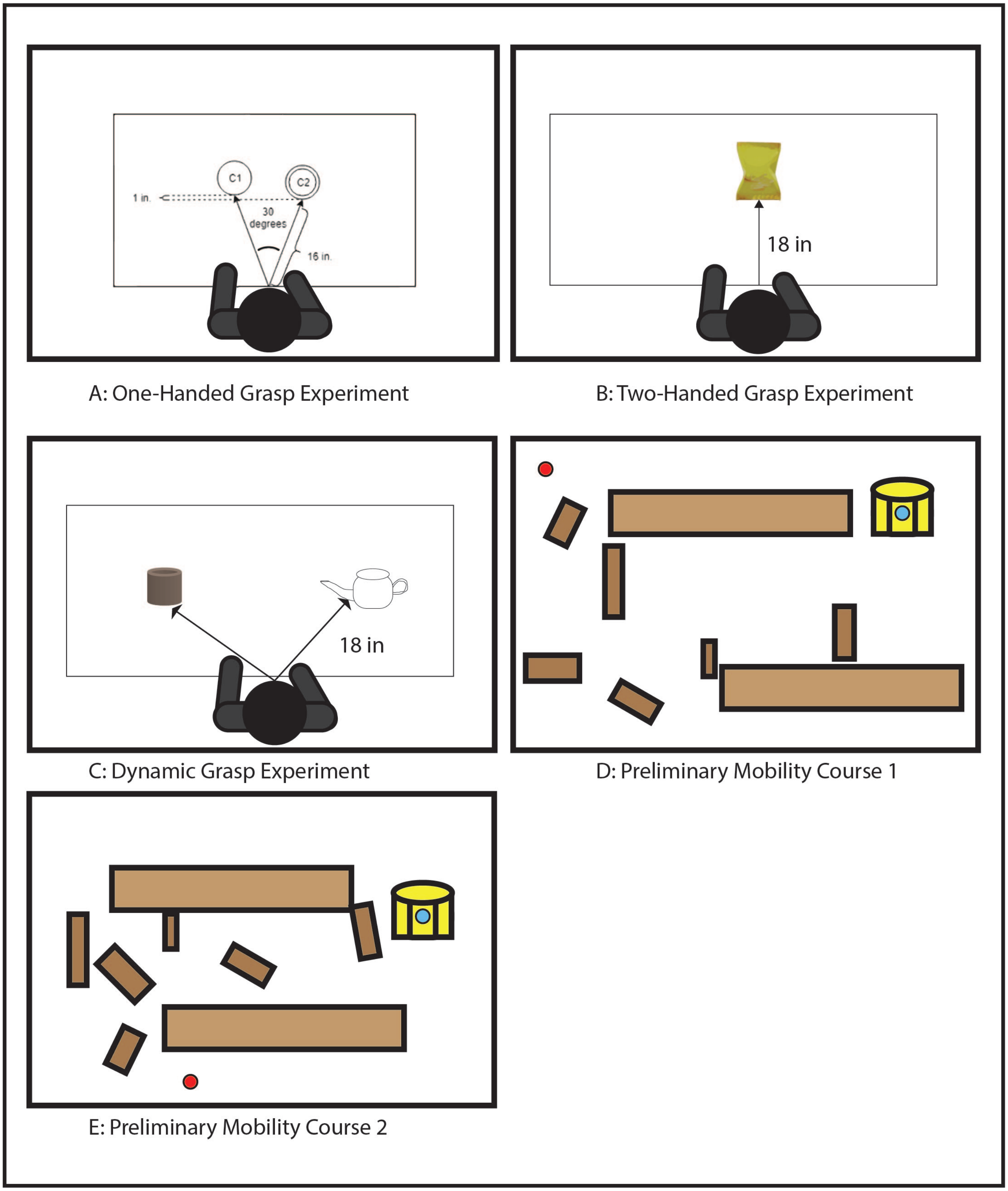

Supplementary Figure 2. Preliminary Experimental Setup. (A): Subjects were asked to grab the closest cylinder. (B): Subjects were asked to grasp the bag with both hands at the same time. (C): Subjects were asked to mime pouring tea into the mug. (D,E): One of the two randomly administered mobility courses for the preliminary sighted subject data. Red dot is starting point, blue dot is endpoint. Both mazes 1 and 2 are the same length and utilize the same obstacles. Subjects were asked to sit in the chair at the end of the course.

Supplemental Figure: Training Obstacle Course

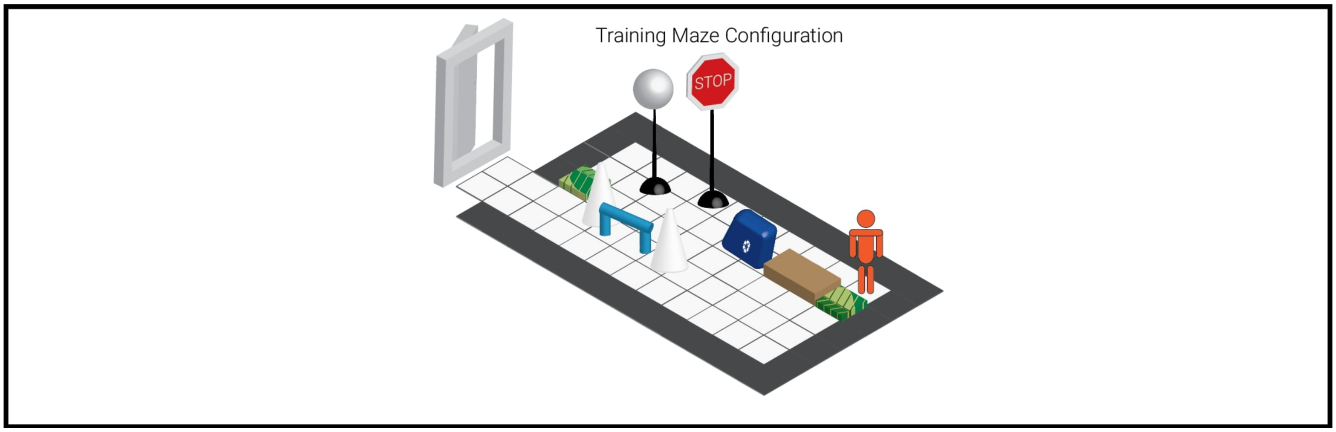

Supplementary Figure 3. Training Maze Configuration. This is the maze that subjects used during training, along with the included audiotape. The training was done in normal office-level lighting. The orange person is the start and the door is the endpoint.

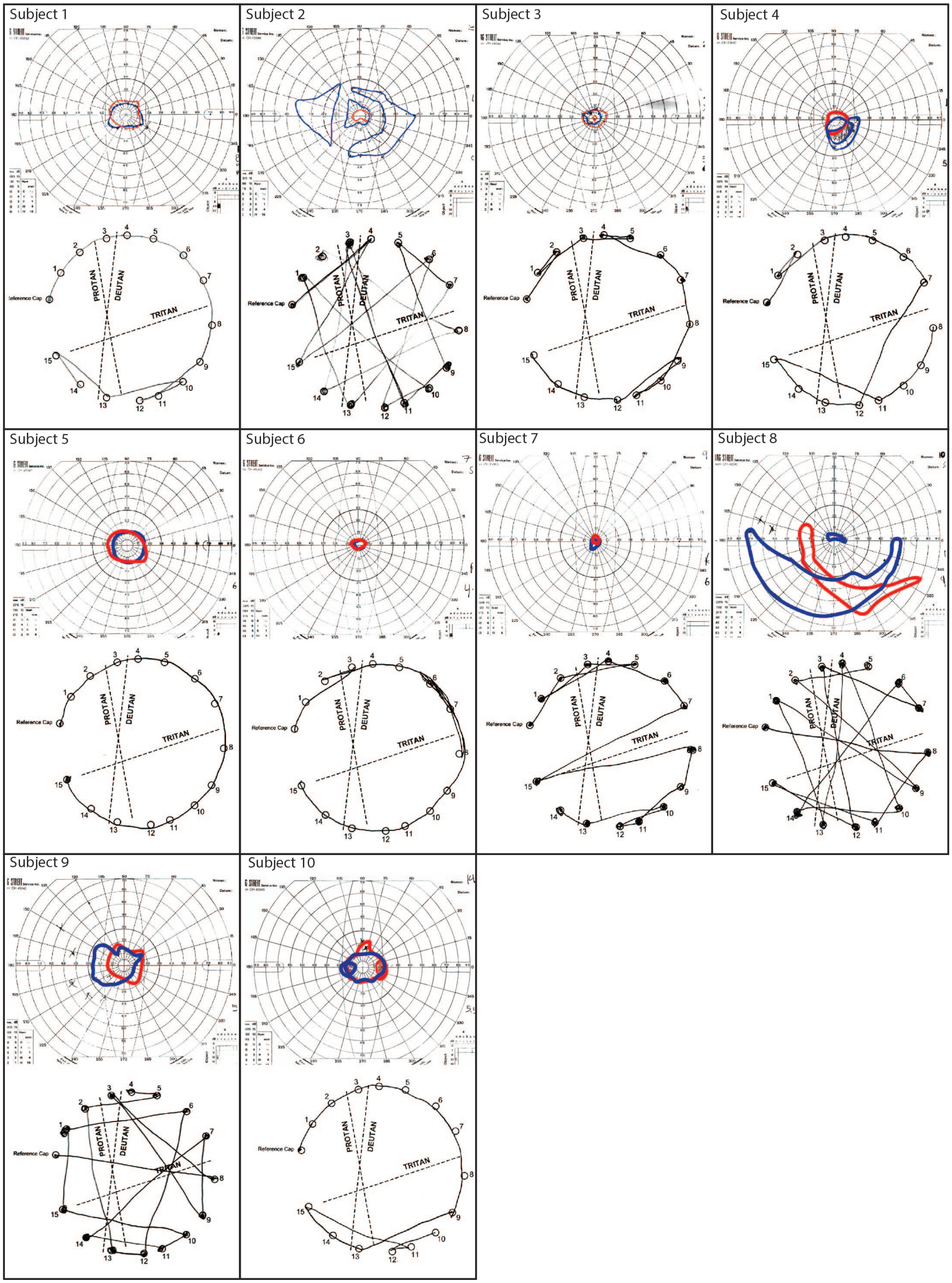

Supplementary Figure 4. Goldmann Visual Fields and Farnsworth D-15 Color Test of RP subjects. Visual fields and color tests were all administered by the same technician on the day of testing. The visual fields are digitally enhanced for clarity, with blue corresponding to the left eye and red corresponding to the right eye visual field. When visual field is reported for eligibility purposes, we look at the largest meridian. D-15 color tests are all binocular.

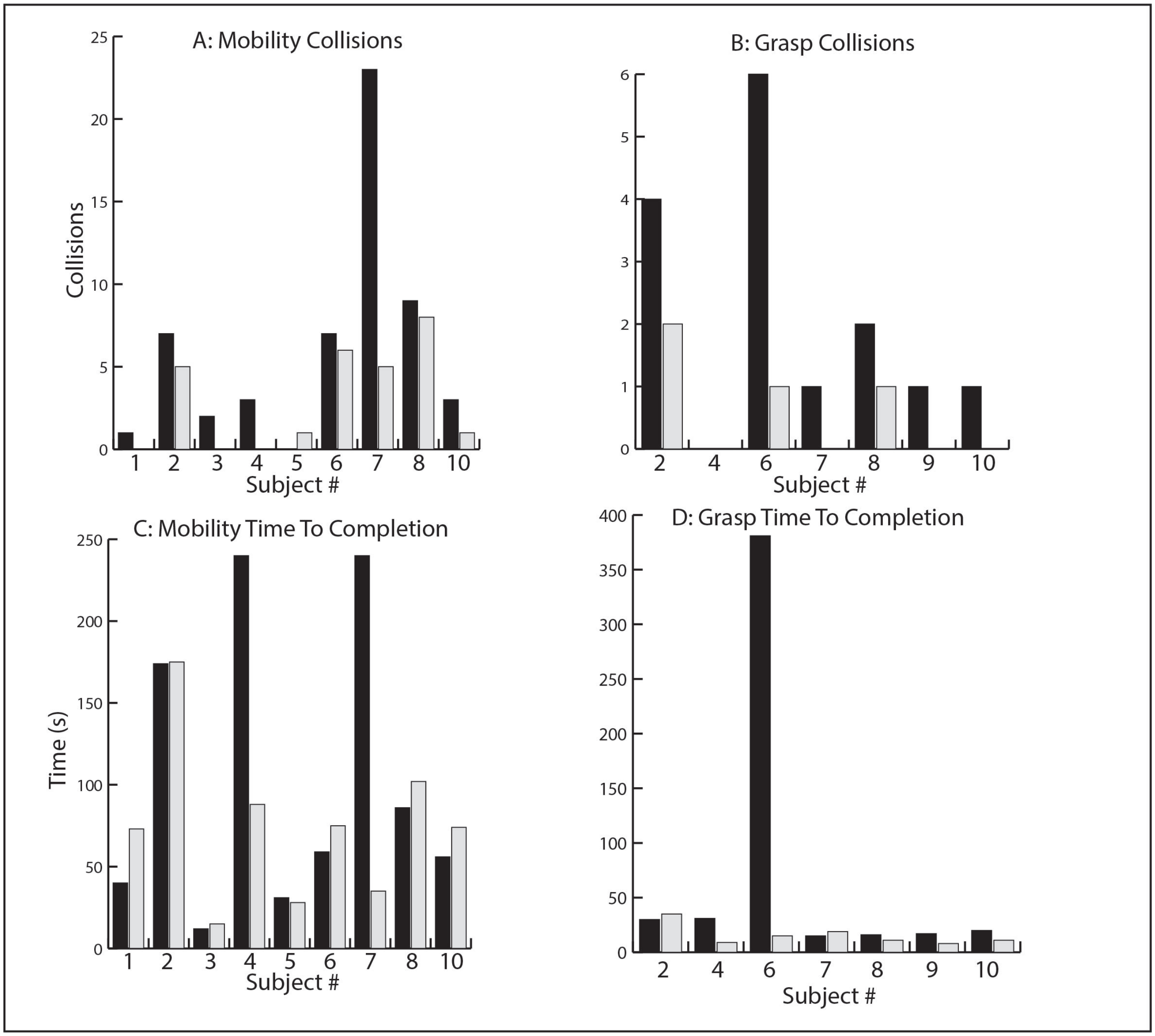

Supplementary Figure 5. Mobility and Grasp Results. Expanded version of figure 2 in the main text, with a bar chart for each experiment. A) Collisions in mobility. B) Collisions in grasp. C) Time to completion for mobility. D) Time to completion for grasp.

Supplementary Data Files 1 and 2, a suggested cover photo, and the video of the encoding are included at the following GitHub link due to size and filetype restrictions:
github.com/aangelopoulos/ARDepthNavigation
Notes on video of encoding: A researcher wearing RP simulation glasses navigated the maze. We used the Hololens' mixed reality recording module to record this video, but this had limitations: lines colored "black" appeared on the recording, but would not show up in real life, as black renderings are transparent in AR. Also, the recording was only taken in the field of view of the device (34 degrees diagonally). Finally, increasing the computational load of the headset by recording data increases the probability of a "glitch," which happens a few times in this video.
